# The Major Stilbene Compound Accumulated in the Roots of a Resistant Variety of *Phoenix dactylifera* L. Activates Proteasome for a Path in Anti-Aging Strategy

**DOI:** 10.3390/cells12010071

**Published:** 2022-12-23

**Authors:** Redouane Benabbes, Sabir Ouahhoud, Mohammed Moueqqit, Mohamed Addi, Christophe Hano, Cédric Delporte, Aminata P. Nacoulma, Véronique Megalizzi

**Affiliations:** 1 Laboratory of Bioresources, Biotechnology, Ethnopharmacology and Health, Faculty of Sciences, Université Mohamed Premier, BV Mohammed VI BP 717, Oujda 60000, Morocco; 2 Laboratory of Improvement of Agricultural Production, Biotechnology and Environment, Department of Biology, Faculty of Sciences, Université Mohamed Premier, Oujda 60000, Morocco; 3 Laboratoire de Biologie des Ligneux et des Grandes Cultures, INRA USC1328, Orleans University, CEDEX 2, 45067 Orléans, France; 4 Microbiology, Bioorganic and Macromolecular Chemistry, Faculty of Pharmacy, Université Libre de Bruxelles, Bvd du Triomphe, 1050 Brussels, Belgium; 5 The Unit Pharmacognosy, Bioanalysis & Drug Discovery (PBDD), Department of Drug Research and Development, Faculty of Pharmacy, Université Libre de Bruxelles, Bvd du Triomphe, 1050 Brussels, Belgium

**Keywords:** *Phoenix dactylifera* L., *Fusarium oxysporum.* sp *Albidinis* (*FoA*), antioxidant activities, anti-tyrosinase activity, proteasome activation, LC-MS/MS analysis, stilbene derivatives

## Abstract

The main objective of the present study is to estimate, through differential analysis, various biological activities of total phenolics content in alcoholic extracts of three date palm varieties sensitive or resistant to *Fusarium oxysporum. sp Albidinis*. Here, stilbene products with antioxidant and bioactive capacities were evidenced in resistant variety Taabdount (TAAR). Furthermore, the methanolic fraction of the TAAR-resistant date palm variety contains a significant product, determined by LC-MS/MS and 1H, 13C NMR, belonging to the family of hydroxystilbenes, which exhibits antioxidant capacities, inhibits the mushroom tyrosinase activity, and activates and exerts a protective effect on hypochlorite-induced damage in 20S proteasome of human dermal fibroblast aged cells. Altogether, the present results indicate that hydroxystilbene present in resistant *Phoenix dactylifera* L. should be studied to understand the way that the stilbene could exert anti-aging ability.

## 1. Introduction

Date palm (*Phoenix dactylifera* L.) is a perennial monocot from the Arecaceae family. This tree is of utmost importance in the lives of Saharan populations of Southern and Southeastern Morocco, both economically and socially. Bayoud disease, which is brought on by the soil fungus *Fusarium oxysporum f.* sp *Albidinis* (FoA) [1], has significantly reduced the yield of Moroccan date palms in recent years [2]. It is estimated that FoA disappeared from two-thirds of Moroccan phoenicicole heritage but continues to destroy between 4.5% to 12% of the highest quality commercial cultivars of date palms [2]. Many efforts have been made to combat this disease and expound some date palm defense mechanisms [3]. The first studies on the possible defense mechanisms of the date palm have highlighted the role of phenolic compounds and antioxidant capacity into tolerance to FoA [4,5]. The biochemical analysis of resistant and sensitive root phenolic compound cultivars was the first biochemical study that elucidated the benefit of defense mechanisms of the date palm. Indeed, the literature indicates that resistant-root cultivars are richer in 5-caffeoyl shikimic acid and its position isomers than of the sensitive-root cultivars [5]. These compounds have effectively shown a potential antifungal activity in vitro against FoA [6].

Natural antioxidants’ great capacity to scavenge free radicals is what is driving their rising popularity in food and medical studies [7,8,9,10]. 

For instance, reactive oxygen species are aggravating factors in cellular injury and aging processes [11] and are thus connected to many diseases. As a result, antioxidant molecules may help human health by preventing degenerative diseases and slowing the consequences of aging [12]. In this study, we are interested in the ability of antioxidants to promote the activation of the 20S-proteasome and protect against oxidative damage of it. It is true that the proteasome contributes to the maintenance of cellular homeostasis by enabling the cell’s process of clearance of damaged proteins and the controlled destruction of short-lived proteins [13]. Therefore, activating the proteasome with antioxidants may prove to be a new anti-aging strategy [14]. Furthermore, this work intends to explore the potential of the phenolic content of resistant roots to FoA as active biological compounds. 

## 2. Materials and Methods

### 2.1. Plant Sample

The study was conducted for each sample with material from 10 adult date palm cultivars (*Phoenix dactylifera* L.) grown from either contaminated or uncontaminated parcels located at the Palmaria Figuig, Southeastern Morocco. The main roots (including their air-breathing roots) run in diameter from 0.5 to 2 cm of susceptible varieties, such as gharas Bouffegous infected (BI) or the uninfected ones (BNI). In addition, the FoA resistant cultivar of Taabdount (TAAR) was collected, and leaves of two medicinal plants were used locally as a phylactic treatment against FoA. Furthermore, rosemary (R, *Rosmarinus officinalis*) and pomegranate (G, *Punica granatum* L.) [15] were also collected in the same uncontaminated parcel whereby they were analyzed and used as the control. Samples were taken during two periods of the year, in September and late January (between 2013 and 2015), and hermetically stored at room temperature. As the samples were acquired from a palmeria, they are duly certified (identification and classification) by Morocco’s Minister of Agriculture [16].

### 2.2. Preparation of the Extracts

After an extensive wash with water, the roots or leaves were dried in the shade, then ground up in a blender. For each sample (BI, BNI, TAAR, R, and G), 10 mL of methanol were added to 1 g of dry material and then left stirring for 4 h at room temperature. Then, they were filtered and evaporated at 37 °C by a rotary-vap. The dry extract was placed in methanol solution at a final concentration of 10 mg/mL or 50 mg/mL (later used to assay biological activities in vitro). Three independent extractions for each sample were performed for the biological activity assays.

### 2.3. Biological Activities

#### 2.3.1. Phenolic Content and Antioxidant Activities

Quantification of total phenolic and flavonoid content

The procedure outlined by Nacoulma et al. was applied to evaluate the total phenolics and total flavonoids content of each extract [17], with a final adaptation of volumes at 200 µL to be used on a microplate reader. The absorbance at 760 nm and 510 nm, respectively, was determined against a methanol blank. Standard calibration curves of gallic acid (0–300 mg/L) (y = 0.0083x + 0.9106, r^2^ = 0.978) and quercetin (0–100 mg/L) (y = 0.0008x + 0.0597, r^2^ = 0.994), respectively, were used.

Determination of DPPH radical scavenging activity and reducing power assay.

Utilizing the 1,1-diphenyl-2-picrylhydrazyl free radical, antioxidant scavenging activity was investigated (DPPH). Using a final adaption of volumes at 300 µL and 275 µL, respectively, in order to employ a microplate reader, the capacity of the extracts to decrease iron (III) was assessed, as described by Nacoulma et al. [17]. In comparison to a methanol blank, the absorbance was measured at 517 nm and 700 nm, respectively. Then, using the following equation, the free radical-scavenging activity of each solution was estimated as a percentage of inhibition:

% inhibition = 100 (A (blank) − A (sample))/A (blank)

A standard calibration curve was obtained using ascorbic acid (0–100 mg/L) as antioxidant standard (y = 0.0014x + 0.0875, r^2^ = 0.998).

#### 2.3.2. FoA Mycelium Growth Inhibitory Assay

With a small modification, the method described by Neri et al. [18] was used to determine the degree to which pure chemicals inhibited FoA mycelium growth. A 7-day-old culture of FoA had five millimeters of mycelial discs placed onto Petri dishes with PDA medium supplemented with the pure chemicals listed above at 50, 75, and 100 µg/mL. The plates were incubated for 5 days at 28 °C. The average diameter of the colony measured at two right angles was used to assess the FoA mycelium growth inhibition. Each treatment consisted of three tests using three different FoA strains: FoA 41818, FoA 41814 (BCCM, Belgium), and FoA Local (Palemeraie of Figuig, Morocco), as well as a negative control using medium that had not been added.

The formula below was used to compute the mycelial growth inhibitions of FoA:

Growth inhibition (%) = (D_c_ − D_t_) D_c_ × 100

where

D_c_: The control colony’s diameter (mm)

D_t_: The treatment colony’s diameter (mm)

#### 2.3.3. Cell Viability and 20S Proteasome Activity

Normal human dermal fibroblasts from a 70-year-old man (NHDF) (Promocell, Heidelberg, Germany) were used whereby cells were placed at a density of 100,000 cells/mL in RPMI 10% supplemented with 10% (*w*/*v*) FBS, 1% (*w*/*v*) L-glutamine, 100 units/mL penicillin, and 100 μg/mL streptomycin. They were kept in white or transparent 96-microwell plates for 24 h at 37 °C in an incubator with a humidified environment of 5% CO_2_. For the proteasome activity assay, as well as for the evaluation of protective effects on hypochlorite-induced damage, cells were then treated with test materials (pure compound from 5 to 50 µg/mL, positive control at 0.5 and/or 1 µM, and methanolic plant extracts from 15 to 50 µg/mL) for 24 h with a maximum 0.3% of DMSO in the final concentration, simultaneously, without changes, regarding the negative control. Investigating the protective effects of TAAR extract or pure compounds on proteasome activity meant that fibroblast cells were later subjected to 50 µM OCl^−^ for 35 min (selected as optimal, without cell toxicity, oxidant-inhibitor of the 20S proteasome condition) [19]. The following experimental stages were completed in accordance with the supplier’s methodology (Proteasome-Glo Chymotrypsin-Like Cell-Based Assay, Promega Corporation, USA): First, a concurrent cell viability experiment employing crystal violet colorimetric or MTT assays that was carried out under absolutely similar conditions was used to normalize this cells-bioluminescence assay. The standard deviations and average fluorescence in triplicate samples from two separate tests (n = 6) are displayed. All measurements and independent experiments (n = 2) were carried out twice.

#### 2.3.4. Inhibitory Effect on Cell-Free Mushroom Tyrosinase

To investigate the effect on the enzyme activity, the test materials (pure antioxidants or methanolic plant extracts) were pre-incubated with enzymes in phosphate buffer at room temperature. In a nutshell, 300 mL of a 5 mM L-DOPA solution at 50 mM phosphate buffer (pH 6.5) with or without test materials in various concentrations were added to a 96-well microplate along with 100 mL of an aqueous solution of mushroom tyrosinase (20 units). For 40 min, the assay mixture was incubated at 25 °C. After incubation, the reaction mixture’s dopachrome production level was measured spectrophotometrically at 492 nm [20]. A standard calibration curve was plotted using gallic acid (0–1000 µg/mL) (y = −0.087 × + 0.45; r^2^ = 0.97). Each independent measurement and experiment (n = 2) was carried out twice.

### 2.4. Analyses, Isolation and Structure Elucidation Procedures

The ground root (20 g) was extracted with 200 mL of methanol, agitated for 24 h at ambient temperature, filtered, centrifuged at 10,000× *g* for 15 min, and then evaporated at 35 °C using a rotary vacuum evaporation apparatus. The crude extract was diluted with 20 mL of distilled water, re-extracted by 2 × 20 mL of ethyl acetate, and then concentrated using a rotary vacuum evaporation system at 35 °C. The main product was purified by preparative HPLC using a Waters C18 column (SymmetryPrep 150 × 19 mm, 7 µm particle size) with the same LC gradient conditions used above and a flow rate of 5 mL/min. The peak at RT was between 38.8 and 40.8 min, collected between 39.2 and 40.2 min; the total volume collection was lyophilized to give 27 mg of the compound. MS and NMR ^1^H and ^13^C then identified it.

Analyses were performed with a rapid resolution LC 1200 series system using a diode array detector (DAD) used for monitoring UV spectra at 320 nm (Agilent Technologies, Santa Clara, CA, USA). Compound separation was performed on a Beckman C18 column (ULTRASPHERE 250 mm × 4.6 mm, 5 µm particle size) using a 47 min gradient of 10 mM ammonium acetate/0.2% formic acid in water (*v*/*v*), pH 2.9 (=solvent A), and 30/70 acetonitrile/methanol mix (=solvent B). The flow rate was 0.8 mL/min, and solvent B was raised from 5 to 35% in 45 min and re-equilibrated during 2 min. In a series with DAD, an ESI-QTOF 6520 series (Agilent Technologies, Santa Clara, CA, USA) was used for high-resolution MS and targeted tandem MS (MS/MS) analyses. Spectra were acquired in negative and high-resolution (4 GHz) acquisition modes.

^1^H NMR and ^13^C NMR spectra were recorded on a Bruker Avance 300 spectrometer operating at 300 MHz. Free induction decays were processed with the MestreNova 5.3.2 NMR suite from MestreLab Research SL.

### 2.5. Statistical Analysis

All independent experiments were performed in triplicate. All data were presented as the mean values ± SD. Using version 6 of the GraphPad Prism program, the results were analyzed using the nonparametric Kruskal–Wallis test together with the Dunn’s multiple comparison test. *p*-Values 0.05 and lower are regarded as significant.

## 3. Results and Discussion

### 3.1. Total Phenolic and Flavonoid Contents of Methanolic Extracts

Due to their redox properties, plant phenolic compounds have been found to have a variety of biological impacts, including antioxidant activity [21]. Table 1 depicts the total phenolic content of methanol root extracts (500 µg/mL) of susceptible varieties *gharas* Bouffegous infected (BI) and uninfected (BNI) by the FoA or resistant as Taabdount (TAAR). Their concentration varied from 171 ± 14 to 253 ± 47 mg of gallic acid eq./g. The highest amount in these compounds was found in the BNI variety, while the lowest concentration came from the methanol root extract of BI. The number of flavonoids in these same extracts (Table 1)) varied from 240 ± 25 to 406 ± 66 mg of quercetin eq./g, and, once again, the highest amount was to the attributed to the BNI variety. There is a loss of approximately 1/3 of the amount of total number of polyphenols and flavonoids between uninfected (BNI) varieties and infected (BI) or resistant (TAAR) varieties to the FoA. Therefore, it is noteworthy to check how the loss of compounds, described as critical to the antioxidant capacity of the plant, may affect the biological activities in which we are interested.

### 3.2. Antioxidant Potential of Methanolic Extracts

**Figure 1:**
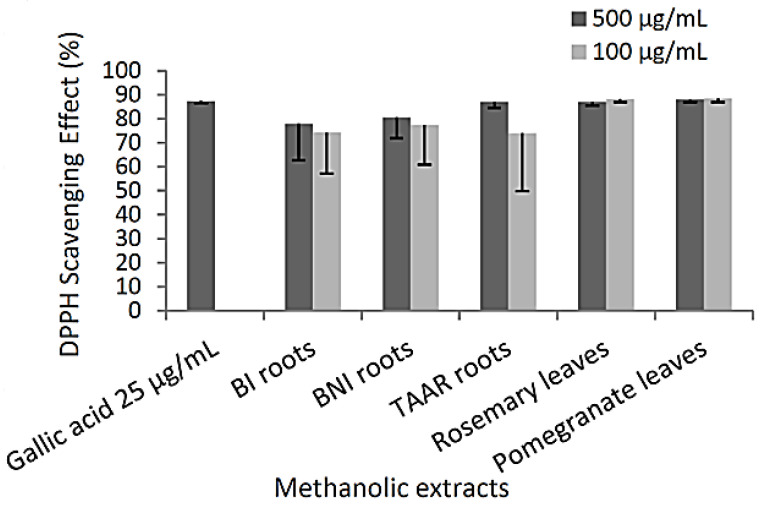
DPPH radical scavenger activity of methanolic extracts. The data are presented as mean (SD) values for each experimental condition (n = 3), and a nonparametric Mann–Whitney test analysis was used to compare the statistical differences between extracts.

DPPH radical scavenging activity is a method widely used to screen the antioxidant activity of plant extracts [22]. This test determines if antioxidant chemicals found in the extracts are capable of scavenging the stable radical species DPPH. The experimental data (Figure 1) reveals that all the methanolic extracts (at 100 or 500 µg/mL) are likely to have the effect of scavenging free radicals with the highest DPPH scavenging activities observed in TAAR roots (87.0% of DPPH inhibition). Antioxidant activity seems not to depend on the presence of the total amount of polyphenolic or flavonoid compounds. However, the better activity of TAAR root methanolic extracts might be due to more hydrogen-donating components contained within the extract.

The reducing power is evaluated by the ability of the methanolic extract to transform the Fe (III) to Fe (II). This ability to reduce Fe (III) may be attributed to the number and to the position of hydroxyl groups present on phenolic compounds and their capacity to donate hydrogen [23]. Table 2 showed the reducing activities of methanolic extracts of our plant samples in comparison with ascorbic acid as a standard. Methanolic extracts of TAAR roots contain high reductions (95 ± 1 mg of ascorbic acid eq./g), while the lowest content was obtained from BNI root extracts (27 ± 2 mg of ascorbic acid eq./g).

A regression analysis was performed to correlate the obtained results (correlation coefficient (R)). Significant linear correlations were found between total phenolic and flavonoid content (R = 0.849, *p* < 0.05) and between DPPH and reducing power assay (R = 0.816, *p* < 0.05), but there were none between the phenolic or flavonoid contents of the methanolic extracts and their antioxidant activities. Finally, all the methanolic extracts exhibited significant antioxidant activities against DPPH radical scavenging activity and reducing power assay, but these antioxidant activities seem unrelated to total phenolic or flavonoid contents. Therefore, these findings imply that a particular type of chemical, rather than the quantity of phenolic or flavonoid compounds found in our diverse extracts, is most likely responsible for the antioxidant activity of the plant extracts under review. In fact, a few phenolic components that were previously described and are highly active individually may also have an impact on the antioxidant activity of plant extracts: Independent of their concentration, the phenolic compounds found in aged red wine may exhibit various antiradical properties connected to their structural aspects [24].

The TAAR FoA-resistant variety shows the highest antioxidant activity among the different varieties of date palms. Referring to the literature, the development of resistance in these particular date palms may be ascribed to an increase in the amount of different positional isomers of caffeoyl shikimic acid [5].

### 3.3. Mushroom Tyrosinase Activity

The mushroom tyrosinase diphenolase activity catalyzes the oxidation of two dopaquinone derivatives leading to melanin synthesis. The results presented in Table 3 show the ability of compounds present in methanolic extracts of TAAR, BI, and BNI to interfere with hydroxylation of l-DOPA through inhibition of the mushroom tyrosinase activity. Furthermore, these results show that methanolic extract of TAAR is statistically more active than extracts of BI (*p* < 0.05) and BNI (*p* < 0.05) on mushroom tyrosinase activity. So, the capacity of palm date roots extracts to inhibit tyrosinase activity seems to follow the same trend as for the antioxidant abilities.

Tyrosinases are primarily associated with skin, eyes, and hair pigmentation. Indeed, tyrosinases are melanogenesis enzymes involved in the first steps of melanin biosynthesis, and melanin is associated with protection against ultraviolet (UV), solar, or gamma radiation, reduced cellular susceptibility, and cell-wall resistance against hydrolytic enzymes [20]. Conversely, an overproduction of melanin may play a role in skin anomalies or more serious disease such as cancer (e.g., Melanoma), and a dopaquinone excess may lead to neurodegenerative disease (e.g., Parkinson’s disease) [20,25]. Moreover, melanogenesis has been reported to produce hydrogen peroxide and other ROS, exposing the human melanocytes to high levels of oxidative stress [26]. Several natural antioxidant compounds (phenolics, flavonoids, and others) obtained from plants inhibited tyrosinase phenolase activity [27,28]. Here, TAAR extract seems to contain a compound(s) susceptible to inhibiting skin aging and melanogenesis.

### 3.4. Proteasome Activity of Methanolic Root Date Palm Extracts

**Figure 2:**
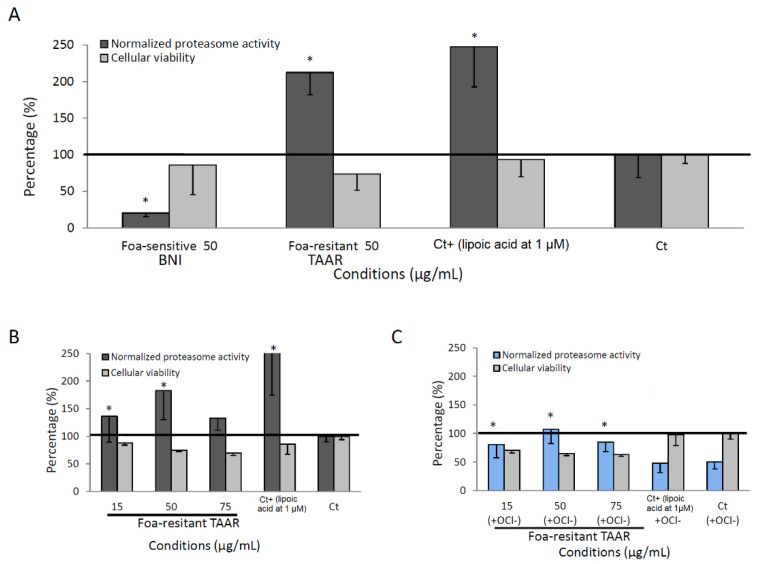
Proteasome activities. (**A**) Determination of the effects of the plant methanolic extracts on the proteolytic activity of the proteasome in human NHDF-aged cells treated with a 50 µg/mL concentration (**B**) or with an increasing concentration of TAAR extract. (**C**) Ability of TAAR methanolic extracts to reverse the proteasome inhibition by the OCl^−^ oxidant. Lipoic acid (1 μM) was used as a reference compound. The data are presented as mean (SD) values for each experimental condition (n = 3), and a nonparametric Mann–Whitney test analysis was employed to compare the statistical difference between extracts: the stars show a significant difference between control (Ct) and other conditions. * Indicates statistically significant differences as compared to controls (*p* < 0.05).

It has been reported that elevated levels of the 20S proteasome lead to increased tolerance to oxidative stress [13]. Therefore, the regulation of proteasome activity by root extracts of BI, BNI, and TAAR, cultivars were investigated. The measured 20S proteasome activity is related to the chymotrypsin-like (CT-L) activity in NHDF-aged cells (see Materials and Methods). As illustrated in Figure 2A, cells treated with 50 µg/mL of methanolic extracts for 24 h exhibited a significant activation of the CT-L for BI and TAAR extracts and an inhibition for BNI without generating more than 30% of cell toxicity. TAAR root extract showed engaging proteasome activation compared with that of 1µM of lipoic acid (Ct^+^) (212% and 248%, respectively). The proteasome is a cylindrical proteinase complex containing a core of four stacked rings responsible for the removal of abnormal degraded (26S) proteasome and oxidatively (20S proteolytic core complex) damaged proteins (Ciechanover, 1998). As presented in Hwang’s work [29], proteasome dysfunction may be a contributor in the aging of human skin. Indeed, aging and replicative senescent cells have been shown to have decreased proteasome activity as well as protein levels of proteasome subunits. The fact that both oxidized and/or damaged cellular proteins accumulated more often in these cells suggests that the ubiquitin–proteasome pathway of protein degradation is implicated in intrinsic aging processes. Figure 2B,C show the ability of TAAR methanolic extract to protect NHDF-aged cells from inhibition of the 20S proteasome activity by OCl^−^ oxidant. Indeed, OCl^−^ oxidant inhibits at 50% the proteasome activity of the control cells (Ct), while the highest 20S-proteasome activator (lipoic acid at 1 µM, Ct^+^) with more than 50% of inhibition activity fails to reverse the inhibition of the 20S proteasome activity by the OCl^−^, whereas all the other concentrations tested (15, 50, and 75 µg/mL) of TAAR methanolic extract seem to reverse this inhibition (0 to 19% of inhibition). These results suggest that TAAR root extracts might contain molecules which could delay skin aging not only by boosting proteasome activity but also by counteracting the inhibiting effects of oxidizing agents. Among these molecules, caffeoyl shikimic acid is most likely to be present, as well as other compounds. Further, LC-DAD-MS (MS) and NMR analyses have been conducted in order to identify the major compounds of the extracts and are hereby discussed in the next section.

### 3.5. Spectrometric Analyses

#### 3.5.1. LC-DAD-MS Analysis of Methanolic Extracts

**Figure 3:**
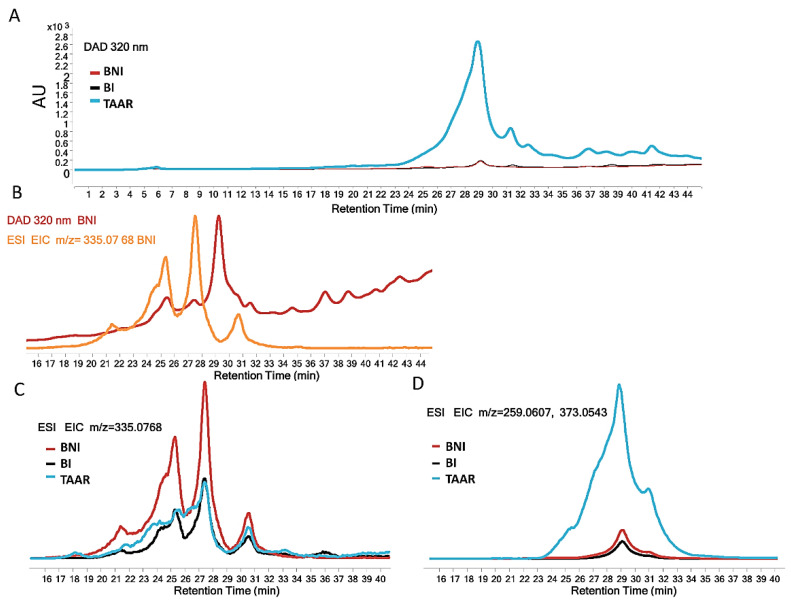
LC-DAD-MS analysis. LC-DAD-MS analysis of BI, BNI, and TAAT methanolic extracts of the root of the date palm, where (**A**) corresponds to DAD chromatograms at 320 nm, (**B**) overlap DAD chromatogram and extracted ion chromatogram (EIC) of the ion 335.0768 targeting all position isomers of caffeoyl-shikimic acid in BNI, (**C**) shows the relative abundance of all position isomers of caffeoyl-shikimic acid and (**D**) shows the relative abundance of 3,3′,4,5,5′-pentahydroxy-trans-stilbene.

The comparative study of date palm roots revealed no qualitative differences, but, instead, semi-quantitative differences (in DAD and MS chromatograms) between the cultivars (Figure 3A,D) were noted to some degree. Indeed, the present results do not allow a link between the resistance of date palms to an increase in the amount of different positional isomers of caffeoyl shikimic acid because the resistant cultivar (TAAR) shows the lowest relative abundance of these compounds (Figure 3C). All three positional isomers of caffeoyl shikimic acid (*m*/*z* = 335.0768 with an error of 1.2 ppm) were identified by their MS/MS spectra (Figure S6) with a fragment at m/z 179.0340, corresponding to a caffeic acid fragment A (C_9_H_7_O_4_) and a fragment at m/z 135.0443, corresponding to the decarboxylation of caffeic acid (C_8_H_7_O_2_). Nonetheless, another compound that does not correspond to caffeoyl shikimic acids (Figure 3C) and whose retention time is 29 min with *m*/*z* = 259.0607 (or 373.0543 for TFA adduct), seems to be clearly distinguished between different cultivars (Figure 3D). The difference in the accumulation of this compound in the roots of sensitive and resistant cultivars (0.135%), with a ratio around 1:35 (from *m*/*z* = 259.06, MS data), could explain the prominent biological activities observed for this root’s methanolic extract.

#### 3.5.2. Determination by MS/MS and NMR of the Major Differential Compound in Date Palm TAAR Resistant Date Palm

In order to determine the structure of the compound present in *Phoenix dactylifera* L. TAAR resistant, liquid–liquid extraction and preparative LC from TAAR extract were performed. Peak with Rt = 29 min was collected and investigated. MS/MS and NMR were then used for structure elucidation. Resulting data were as follows: 1H NMR (300 MHz, (CD3)2SO) δ = 6.08 ppm (t, J = 2 Hz, H4′), 6.33 ppm (d, J = 2 Hz, H2′ and H6′), 6.47 ppm (s, H2 and H6), and (CH=CH) 6.55 ppm (d, J = 16.2 Hz, Ha), 6.72 ppm (d, J = 16.2 Hz, Hb). 13C NMR (75 MHz, (CD3)2SO) δ = 158.73 ppm (C3′ and C5′), 144.85 ppm (C3 and C5), 139.4 ppm (C1′), 129.1 (C4), 124.9 ppm (C1), 124.7 ppm and 123.9 ppm (CH=CH), 105.4 ppm (2 CH, C2 and C6), and 104.1 ppm (2 CH, C2′ and C6′). NMR signals confirmed the presence of the double bond in trans (δ = 6.55 ppm), phenolic groups, and di- and tri-substituted aromatic rings: ESI-QTOF HRMS/MS observed m/z: 259.0607 [M-H]^−^ (100%), and major fragments (MS/MS) 241.0499 [M-H-H_2_O]^−^, 217.0517 [M-H-C_2_H_2_O]^−^, 213.0551 [M-COH_2_O]^−^, 199.0389 [M-C_2_H_4_O_2_]^−^, 175.0393 [M-C_4_H_4_O_2_]^−^, 171.0455 [M-H-C_3_H_4_O_3_]^−^ with, for all observed m/z, an error below 6 ppm compared to calculated m/z. The [M-H]^−^ of the compound was 259.0607 m/z with two main fragments at 217.0517 [C_12_H_9_O_4_]^−^ and 175.0408 [C_10_H_7_O_3_]^−^, corresponding to the consecutive losses of C_2_H_2_O, characteristic of stilbenoids [30]. A comparison of the observed spectral data with those reported in the literature [31,32] confirms the results and the presence of 3,3′,4,5,5′-pentahydroxy-trans-stilbene compound. Thus, the aromatic region of the ^1^H and ^13^C NMR spectra (Figures S7 and S8) exhibited features that were virtually the same as those observed for the stilbene 3,3′,4,5,5′-pentahydroxy-trans-stilbene [32], as was further confirmed by the MS/MS fragmentation pattern of other compounds (Figure S5), which is consistent with the proposed structure.

### 3.6. The Stilbene Inhibition of FoA Mycelium Growth Is Associated with Date Palm Resistance to FoA

**Figure 4:**
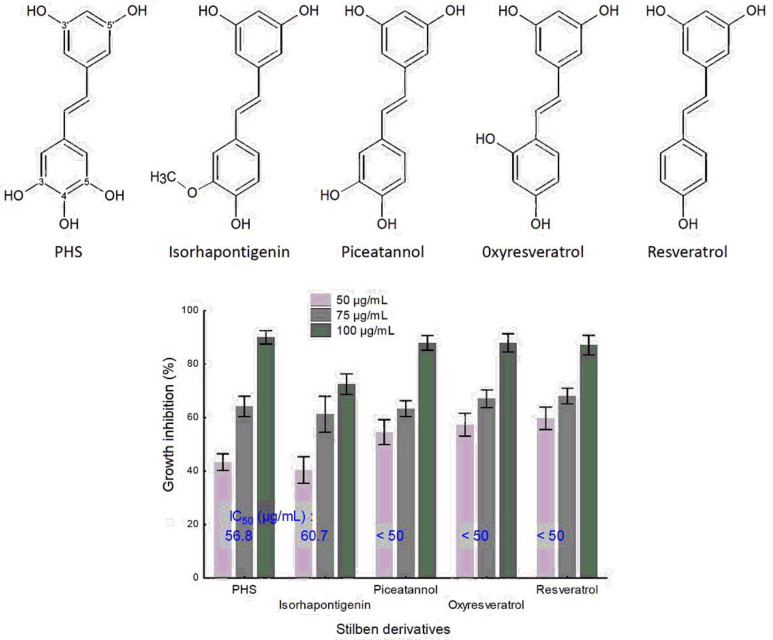
Inhibitory effect of five pure stilbenoids on FoA mycelium growth. Determination of the effects of piceatannol, oxyresveratrol, resveratrol, isorhapontigenin, and 3,3′,4,5,5′-pentahydroxy-trans-stilbene (PHS) on the growth of three strains of FoA. The data are presented as mean (SD) values for each experimental condition (n = 3).

The effect of the five hydroxy-stilbenoid derivatives was evaluated at three different concentrations (50, 75, and 100 µg/mL) on three strains in vitro FoA mycelium growth. As shown in Figure 4, for all tested compounds, as the concentration increased, mycelium growth inhibition increased as well. For instance, PHS at 50, 75, and 100 µg/mL inhibits FoA mycelium growth by 43, 64, and 90%, respectively, with an IC_50_ value of 56.8 µg/mL. However, according to the tested compound, IC_50_ values varied from <50.0 to 60.7 µg/mL (for resveratrol, oxyresveratrol, piceatannol, PHS, and isorthapontigenin, respectively), suggesting that hydroxyl and/or methoxy-substitutions of the B-ring of the basic chemical structure of stilbene is able to induce inhibition of FoA mycelium growth.

Although mechanisms of stilbene toxicity towards pathogenic fungus cells are not well understood, this class of secondary metabolites seems to target crucial metabolic or structural components of the cell. Accordingly, several studies suggest that toxicity of several stilbenoids can be due to their capacity to penetrate lipophilic membranes and to disorganize/disrupt cell membrane integrity and structure [33].

### 3.7. Proteasome Activity of Pure Stilbene Compounds

**Figure 5:**
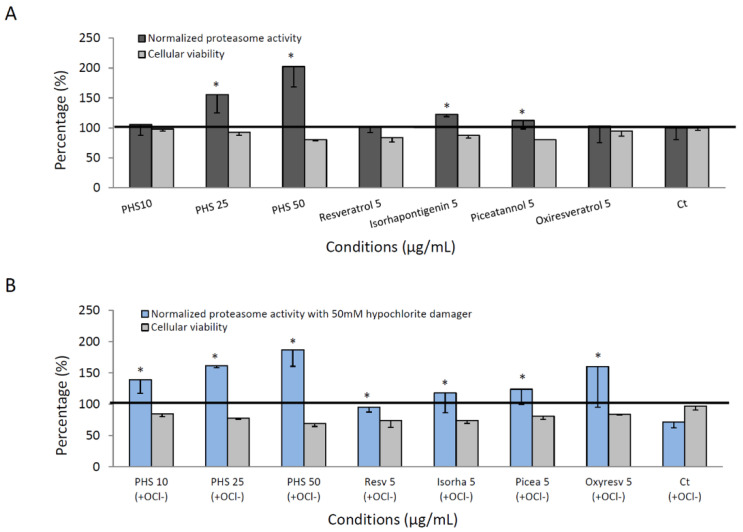
Activation and the protective effect on the proteasome of five pure stilbenoids. (**A**) Determination of the effects of piceatannol, oxyresveratrol, resveratrol, isorhapontigenin, and 3,3′,4,5,5′-pentahydroxy-trans-stilbene (PHS) on the proteolytic activity of the proteasome in human NHDF-aged cells. (**B**) Ability to purify PHS or commercial compounds to reverse the proteasome inhibition by the OCl^−^ oxidant. The data are presented as mean (SD) values for each experimental condition (n = 3), and a nonparametric Mann–Whitney test analysis was used to compare the statistical difference between pure compounds: the stars show a significant difference between control (Ct) and other conditions. * Indicates statistically significant differences as compared to controls (*p* < 0.05).

In an additional study, we analyzed the activation and the protective effect on the proteasome of five stilbenoids. These are all hydroxy-stilbene including resveratrol (3,4′,5-trihydroxy-trans-stilbene,), oxyresveratrol (3,3′,5,5′-tetrahydroxy-stilbene), piceatannol (3,3′,4′,5-tetrahydroxy-stilbene), and isorhapontigenin (3,4’,5-Trihydroxy-3’-methoxy-trans-stilbene) and 3,3′,4,5,5′-pentahydroxy-trans-stilbene (PHS) previously isolated. Figure 5A shows an increase in proteasome activity for PHS (25 and 50 µg/mL) as well as isorhapontigenin (5 µg/mL) when compared to the control cell condition. Since all these four commercial tested hydroxy-stilbenes were shown to be cytotoxic at concentration higher than 5 µg/mL, proteasome activity was tested at this concentration, which has only a slight incidence on cell viability (less than 30% inhibition), and, as shown in Figure 5A, only moderate but significant induction in proteasome activity was observed for isorhapontigenin and piceatannol (122 and 112% respectively) as compared to the control. The result suggests that a loss of functional groups (hydroxy or methoxy) in C3 and C5 position, which differentiates PHS from the other related hydroxy-stilbenoids, contribute to the attenuation of proteasome activity. Significantly, whereas OCl^−^ oxidant (hypochlorite damager) inhibits at 30% proteasome activity of the control cells (Ct), all hydroxystilbenoids were able to protect the proteolytic activities of the 20S proteasome within human NHDF-aged cells exposed to hypochlorite damage (Figure 5B).

Hydroxystilbene derivatives are poorly represented in the Arecaceae family; some of them have been isolated from seeds of Syagrus romanzoffiana [34], Aiphanes aculeata [35], or in roots of Phoenix dactylifera L. [36]. While other hydroxy stilbenes were found in Phoenix dactylifera L. [36], 3,3′,4,5,5′-pentahydroxy-trans-stilbene was reported for the first time in the date palm. This overexpression of stilbene in some cultivars is not surprising because of the evidence whereby induction of biosynthesis of stilbene phytoalexins and their accumulation fall in the category of active defense mechanisms of hosts against biotic stress [37]. The hydroxylated stilbenes are known to exhibit pronounced antioxidant activity [31,32]. Thus, the highest antioxidant activity exhibited by TAAR methanolic extract may certainly be explained by the presence of the high amount of 3,3′,4,5,5′-pentahydroxy-trans-stilbene. One endeavor by Lam et al. [38] has reported 3,3′,4,5,5′-pentahydroxy-trans-stilbene with therapeutic potential as hypoglycemic agents, although it could very well be potent against a variety of diseases. Indeed, various hydroxylated stilbenes or stilbenoids have shown HIV-1 inhibitory activity [39], anti-inflammatory [40], and even anti-cancer activity [41,42]. Although many in-vitro studies have highlighted the cytoprotective effect of a polyphenol diet against oxidative stress or cell death, their ability in a high concentration or in the presence of metal ions to form hydroxyl radical can also display a prooxidant activity of polyphenol [43]. This could explain the interesting results reported in the study of Li and al., demonstrating the PHS-induced apoptosis in colorectal tumor cells via oxidative stress [44].^.^

In this regard, the review of Bekhet and Eid highlights the duality of effects of antioxidants. A strict management of antioxidant doses is needed to control the ROS effects and to target specific redox pathways involved in cancer progression without disrupting the overall redox balance in normal cells in order to avoid an enhanced cytotoxic effect of the therapy [45].

Furthermore, it is commonly used in cosmetics and dermatology as an epidermis and dermis cellular rejuvenator or antiwrinkle agent [46]. A comprehensive review [47,48] highlights the role played by stilbene polyphenols in molecular mechanisms of defense against oxidative stress, emphasizing the crucial role of the nuclear factor-erythoid-2-related factor-2 (Nrf2) in the cellular defense process in mammals and aging-related diseases. These uses might be related to the proteasome activation in skin cells demonstrated in our study.

## 4. Conclusions

The roots of TAAR, a resistant variety of *Phoenix dactylifera* L., produced a significant amount of 3,3′,4,5,5′-pentahydroxy-trans-stilbene compared to those of uninfected or infected varieties. Moreover, our spectrometric and semi-quantitative analysis showed that it is also the major product present in large amounts in methanolic extract of the resistant variety. Therefore, the main biological activities that we have identified in this extract may be correlated with the presence of this stilbene derivative. Overall, 3,3′,4,5,5′-pentahydroxy-trans-stilbene appears to be a potential constitutive defense weapon of *Phoenix dactylifera* L. in the fight against Bayoud disease. Finally, 3,3′,4,5,5′-pentahydroxy-trans-stilbene demonstrated the ability to exert a new cellular protective role towards proteasome preservation and activation, which may contribute significantly to the field of proteasome-related diseases.

## Figures and Tables

**Table 1:** Total phenolic and flavonoid contents of plant methanolic extracts.

Methanolic Extract (500 µg/mL)	Polyphenols Contents (mg of GAE/g)			Flavonoid Contents (mg of QE/g)		
BI roots	171	±	14	245	±	104
BNI roots	253	±	47	406 *	±	66
TAAR roots	204	±	14	240 *	±	25
Rosemary leaves	117	±	22	207	±	62
Pomegranate leaves	175	±	0	207	±	4

Each value in the table is represented as mean ± SD (n = 3). The data are presented as mean (SD) values for each experimental condition (n = 3), and a nonparametric Mann–Whitney test analysis was used to compare the statistical differences between extracts. * Indicates statistically significant differences as compared to controls (*p* < 0.05).

**Table 2:** Reducing power of plant methanolic extracts.

Methanolic Extract (500 µg/mL)	Reducing Power Assay (mg of AAE/g)		
BI roots	43	±	5
BNI roots	27 *	±	2
TAAR roots	95 *	±	1
Rosemary leaves	73	±	2
Pomegranate leaves	165	±	15
Gallic acid (25 µg/mL)	1026	±	144

Each value in the table is represented as mean ± SD (n = 3). The data are presented as mean (SD) values for each experimental condition (n = 3), and a nonparametric Mann–Whitney test analysis was used to compare the statistical differences between extracts. * Indicates statistically significant differences as compared to controls (*p* < 0.05).

**Table 3:** Tyrosinase inhibitory activity of methanolic extracts of date palm roots.

Methanolic Extract (500 µg/mL)	Inhibition of Tyrosinase Activity (mg of GAE/g)		
BI roots	27	±	11
BNI roots	11	±	4
TAAR roots	87	±	26

Each value in the table is represented as mean ± SD (n = 2). The data are presented as mean (SD) values for each experimental condition (n = 3), and a nonparametric Mann–Whitney test analysis was used to compare the statistical differences between extracts.

## Data Availability

Not applicable.

## Supplementary Materials

The following supporting information can be downloaded at: https://www.mdpi.com/article/10.3390/cells12010071/s1. The complete and original images from the MassHunter software DAD, MS/MS, EIC chromatograms and ^1^H, ^13^C NMR spectra of compounds are shown in Figures S1–S8.
