# Supplementary material for: The Major Stilbene Compound Accumulated in the Roots of a Resistant Variety of Phoenix dactylifera L. Activates Proteasome for a Path in Anti-Aging Strategy
Source: Cells. 2022 Dec 23;12(1):71. doi: 10.3390/cells12010071 (PMC9818208; doi:10.3390/cells12010071)
Supplement: Supplementary file 1 [file cells-12-00071-s001.zip › cells-2028840-supplementary.pdf]

The Major Stilbene Compound Accumulated in the Roots of a Resistant Variety of *Phoenix dactylifera* L. Activates Proteasome for a Path in Anti-Aging Strategy

Redouane Benabbes [1], Sabir Ouahhoud [1], Mohammed Moueqqit [1], Mohamed Addi [2], Christophe Hano [3,*], Cédric Delporte [4], Aminata P. Nacoulma [5] and Véronique Megalizzi [4,5]

[1] Laboratory of Bioresources, Biotechnology, Ethnopharmacology and Health, Faculty of Sciences, Université Mohamed Premier, BV Mohammed VI BP 717, Oujda 60000, Morocco
[2] Laboratory of Improvement of Agricultural Production, Biotechnology and Environment, Department of Biology, Faculty of Sciences, Université Mohamed Premier, Oujda 60000, Morocco
[3] Laboratoire de Biologie des Ligneux et des Grandes Cultures, INRA USC1328, Orleans University, CEDEX 2, 45067 Orléans, France
[4] Microbiology, Bioorganic and Macromolecular Chemistry, Faculty of Pharmacy, Université Libre de Bruxelles, Bvd du Triomphe, 1050 Brussels, Belgium
[5] The Unit Pharmacognosy, Bioanalysis & Drug Discovery (PBDD), Department of Drug Research and Development, Faculty of Pharmacy, Université Libre de Bruxelles, Bvd du Triomphe, 1050 Brussels, Belgium
* Correspondence: hano@univ-orleans.fr

Supplementary Material

<u>MS parameters</u>:

Gas temperature of 325 °C; drying gas of 11 L/min; nebulizer pressure gauge of 50 psi; capillary voltage of 4000 V; Fragmentor: 175 V; Fixed collision energy (CE): 20-30-50 V (for targeted MS/MS); MS scan range and rate: 100-1000 at 3 spectra/s; MS/MS scan range and rate: 50-1000 m/z at 3 spectra/s. Data were acquired by the Mass Hunter Acquisition® software (Version B.04, patch 3) and analyzed by the Mass Hunter Qualitative Analysis (Version B.06) software's.

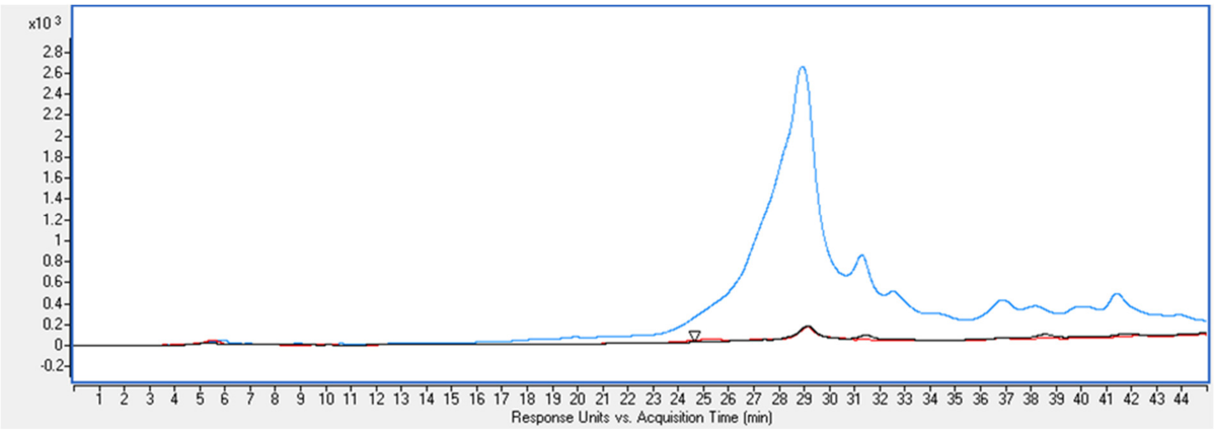

Figure S1. DAD chromatogram of BI, BNI and TAAT methanolic extracts of date palm roots detection was carried out at 320 nm (BNI in red line, BI in black line and TAAR in blue line).

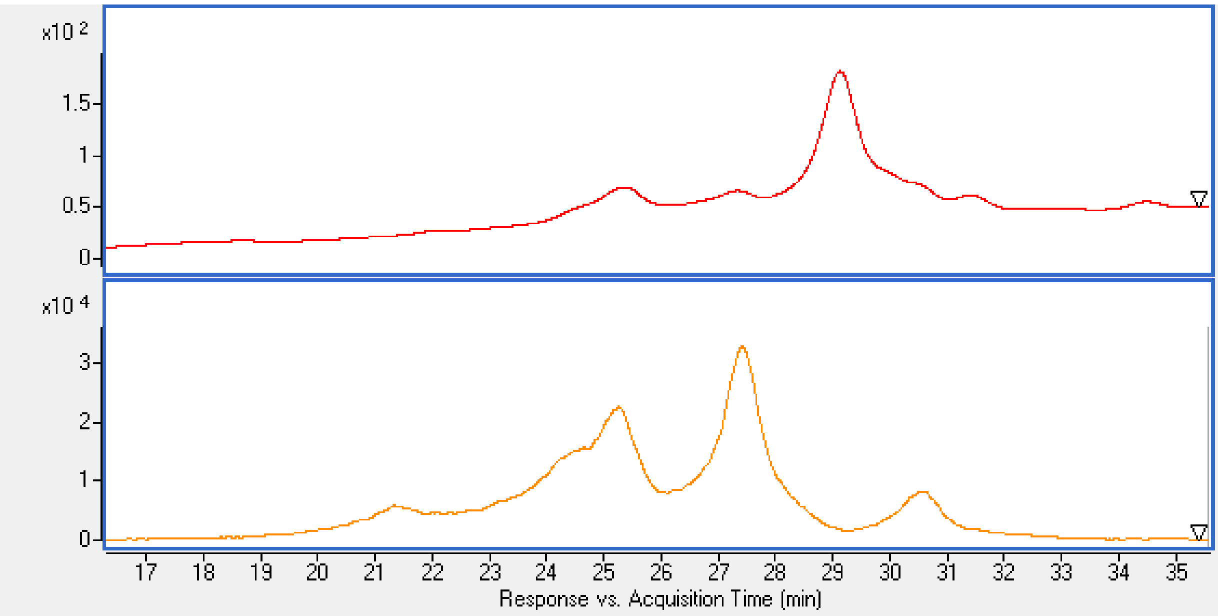

Figure S2. DAD chromatogram (red) and ESI MS (EIC, extracted ion chromatogram in orange), targeting all position isomers of caffeoyl-shikimic acid in BNI.

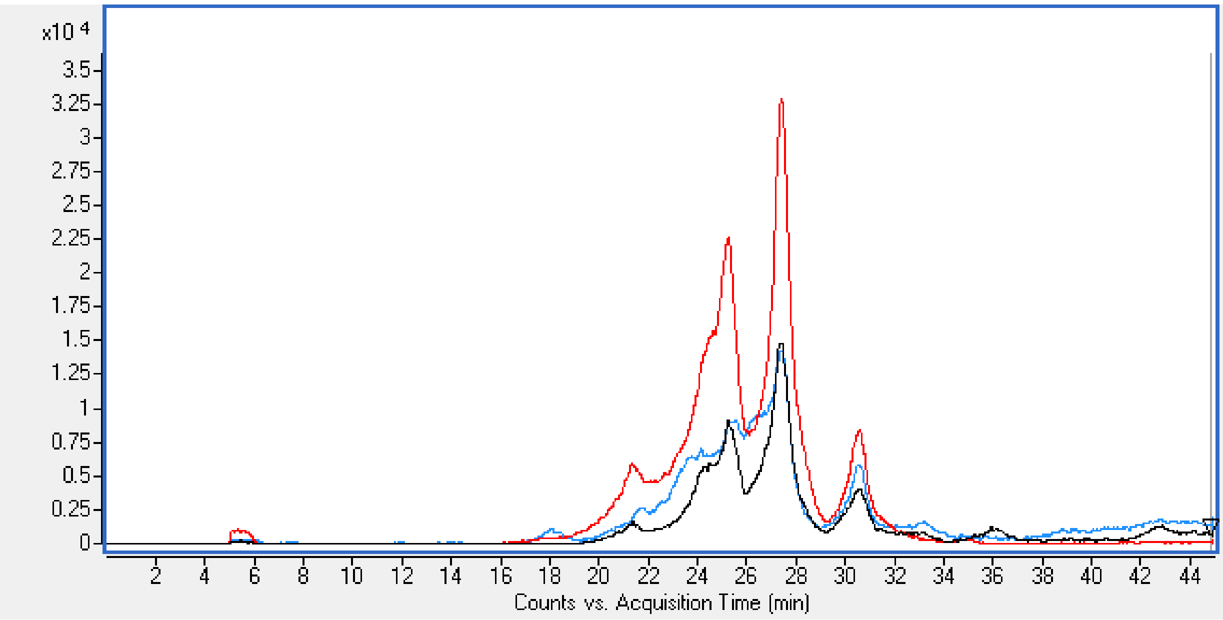

Figure S3. ESI MS (EIC, extracted ion chromatogram) showing the relative abundance of all position isomers of caffeoyl-shikimic acid in BNI (red) BI (black) and TAAR (Blue) methanolic extracts of date palm root.

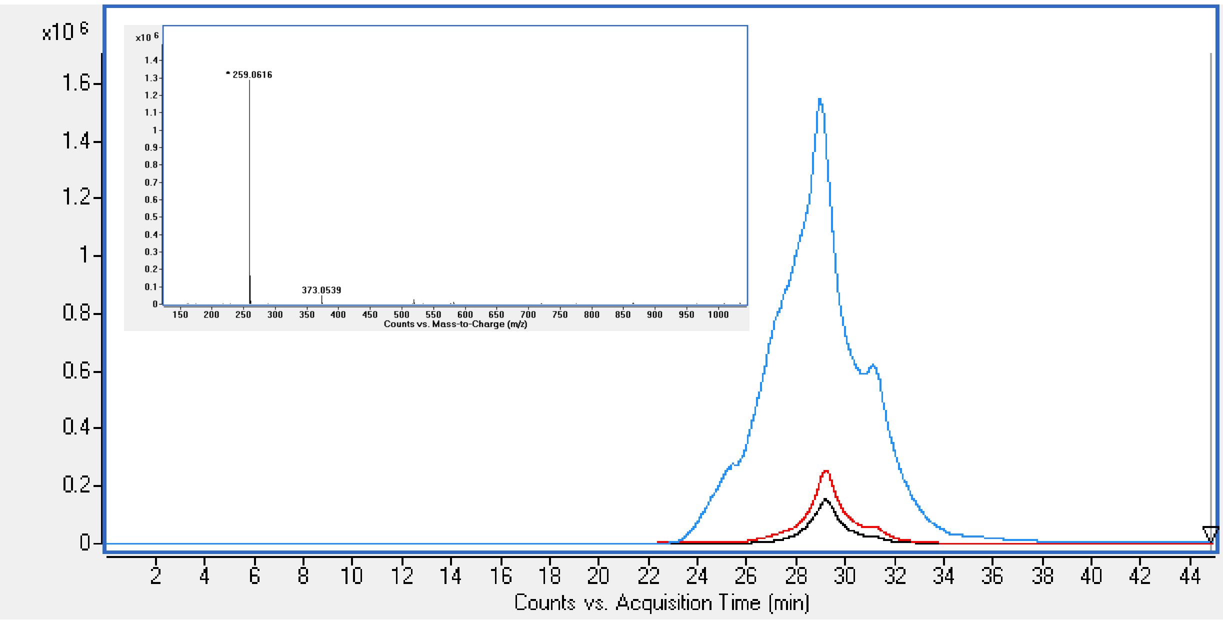

Figure S4. ESI MS (EIC, extracted ion chromatogram) showing the relative abundance of 3,4,5,3′,5′- pentahydroxy-trans-stilbene in BNI (red) BI (black) and TAAR (Blue) methanolic extracts of date palm root. MS data in the inset (m/z = 259.0607 or 373.0543 for TFA adduct).

A)

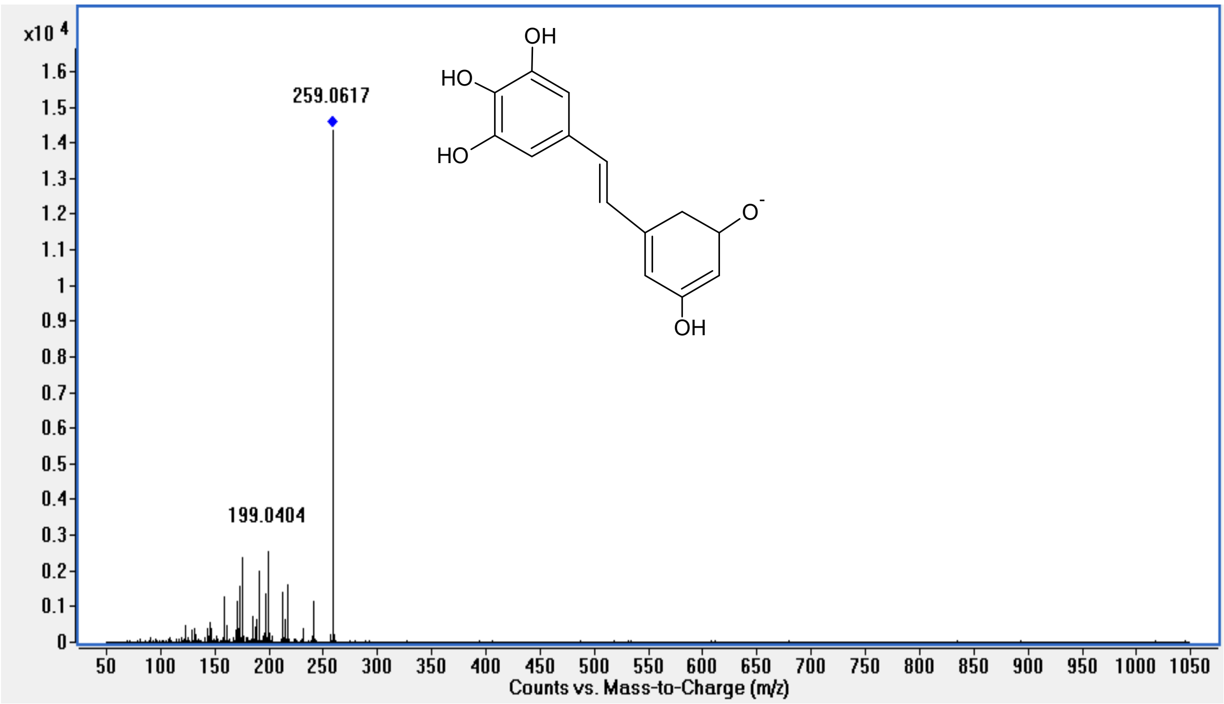

B)

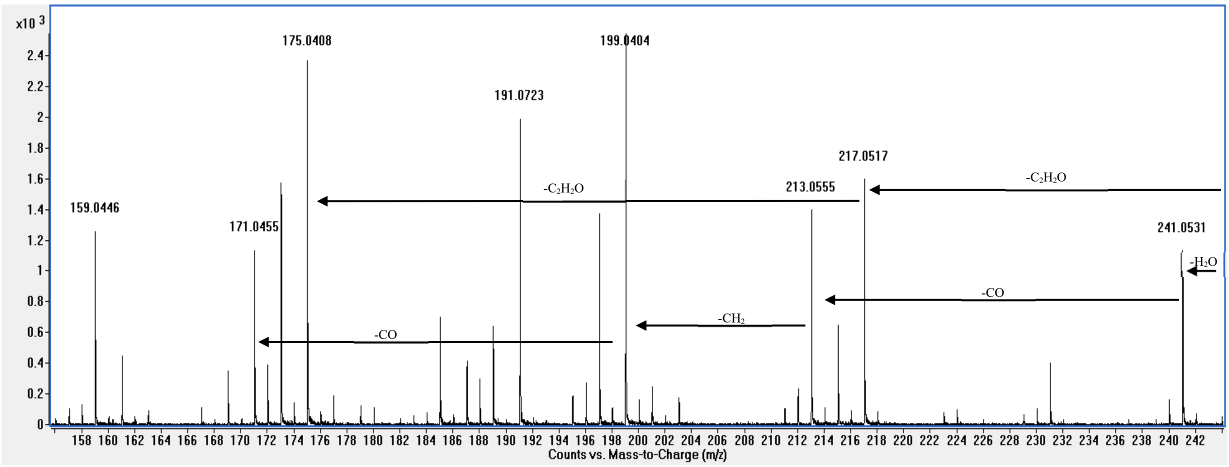

Figure S5. (**A**) MS/MS spectra of peak with the Rt=29 min in BNI, BI and TAAR methanolic extracts of date palm root: parent ion at m/z 259 in negative ion mode. (**B**) Enlarged showing fragmentation and the characteristic product ions identified, m/z 241 corresponding to a dehydrated fragment, m/z 217.0517 ($C12H9O4^-$) and m/z 175.0408 ($C10H7O3^-$) corresponding to the consecutive losses of C2H2O, characteristic of stilbenoids.

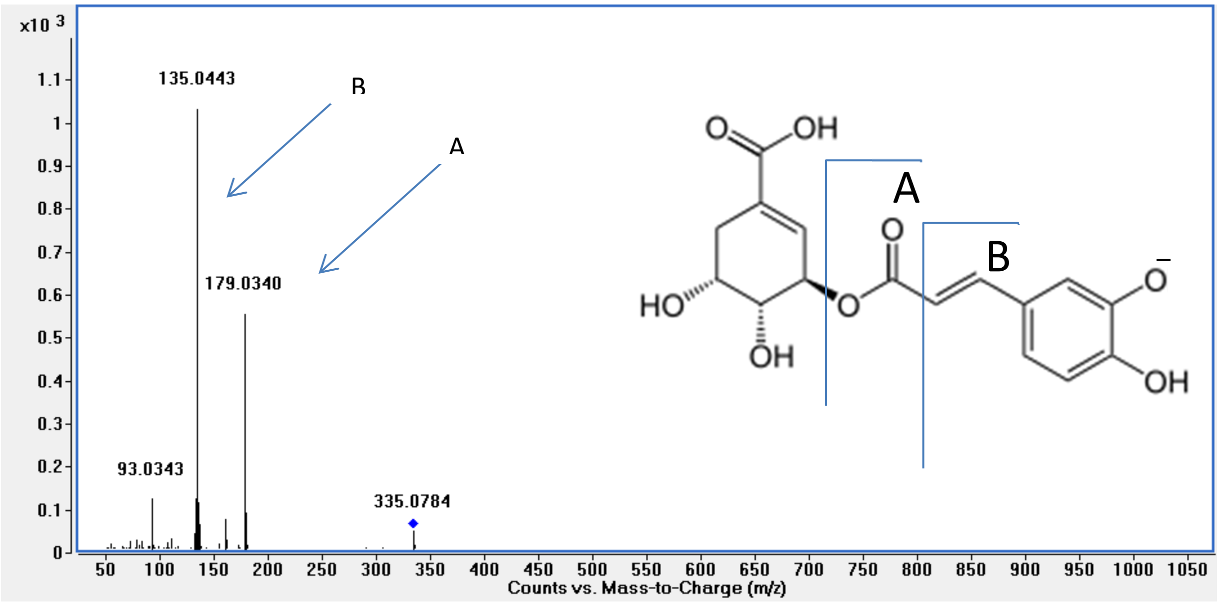

Figure S6. MS/MS spectra of all position isomers of caffeoyl-shikimic acid in BNI, BI and TAAR methanolic extracts of date palm root: parent ion at m/z 335, m/z 179 corresponding to a caffeic acid fragment A (C9H7O4), and m/z 135 corresponding to a decarboxylation of caffeic acid fragment B (C8H7O2) in negative ion mode.

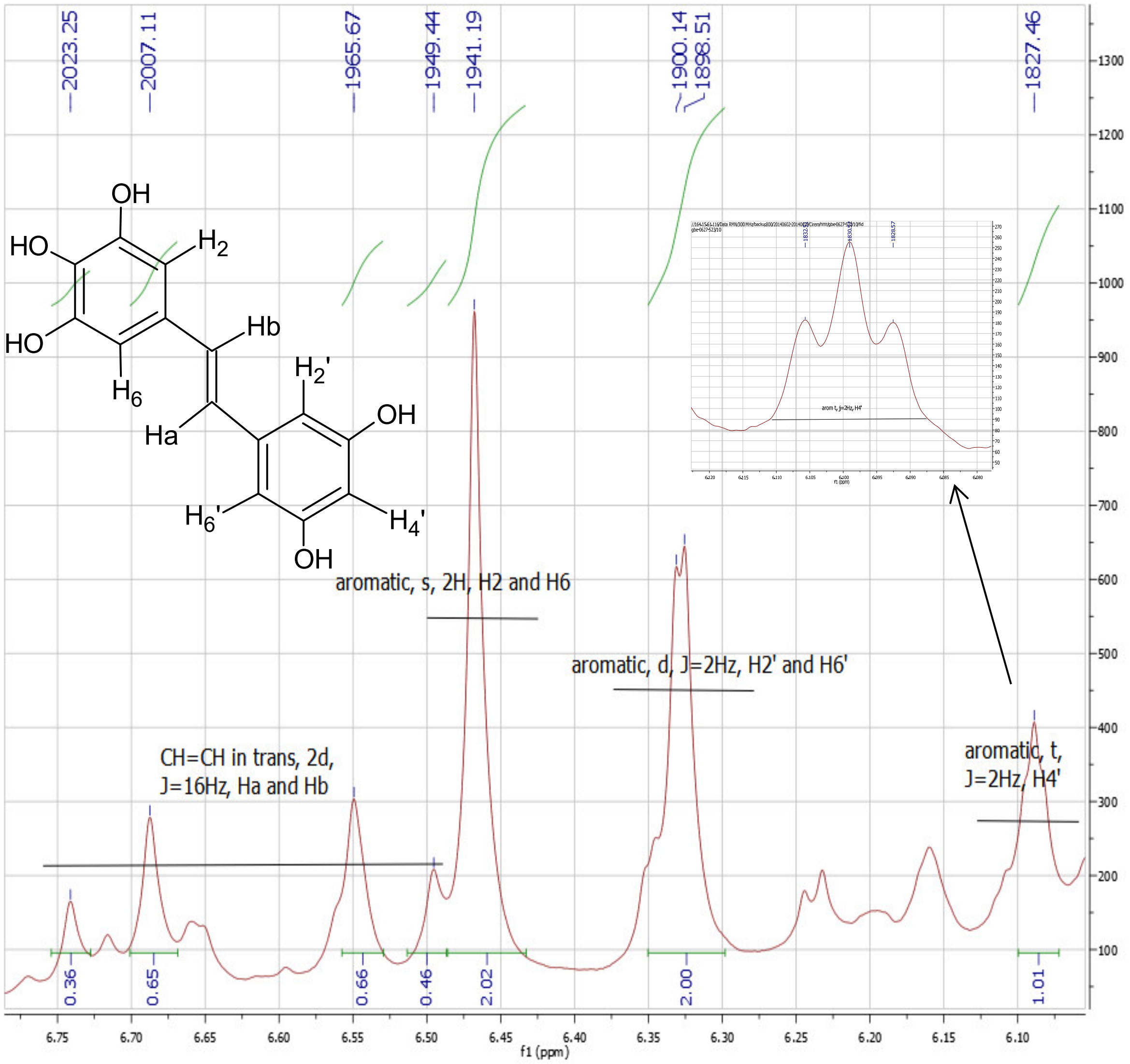

Figure S7. Aromatic region of the ^{1}H NMR spectrum of 3,4,5,3′,5′- pentahydroxy-trans-stilbene.

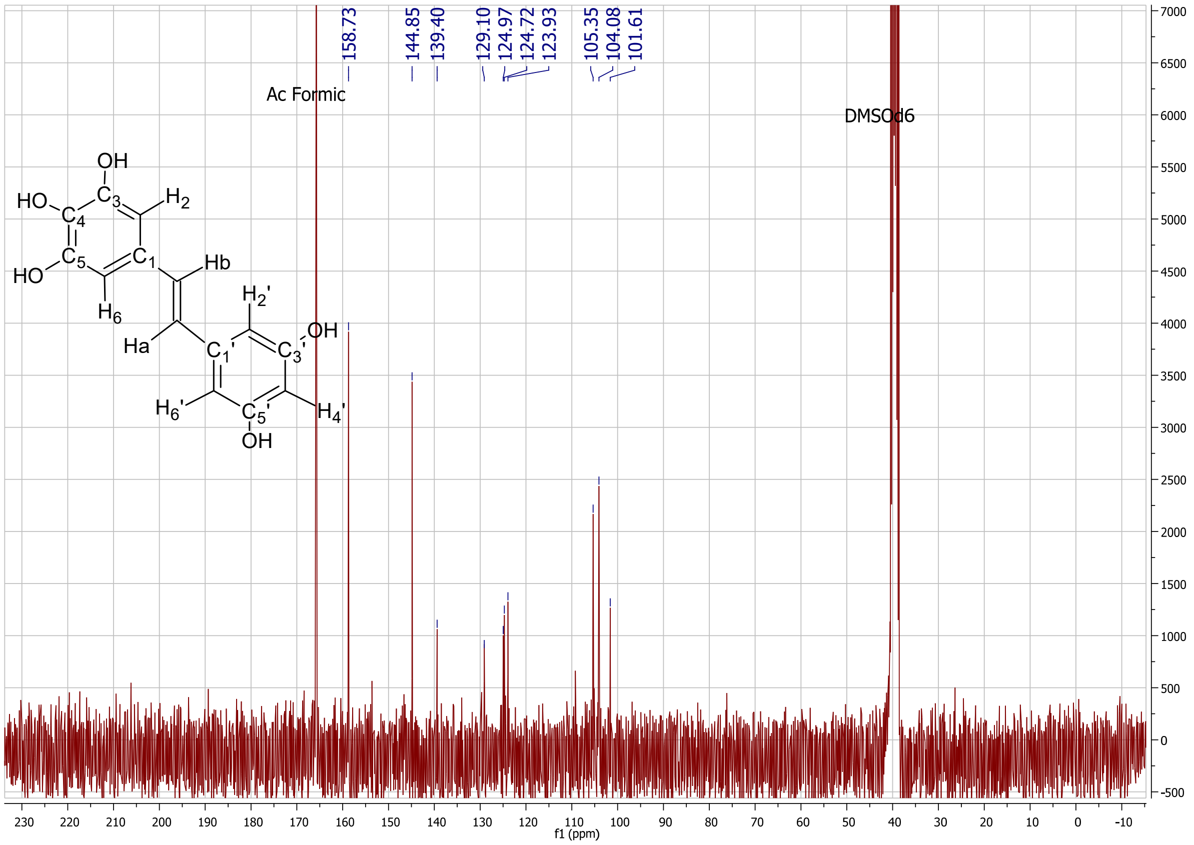

Figure S8. ^{13}C NMR spectrum of 3,4,5,3′,5′- pentahydroxy-trans-stilbene.
